# High Fluence Chromium and Tungsten Bowtie Nano-antennas

**DOI:** 10.1038/s41598-019-49517-y

**Published:** 2019-09-10

**Authors:** Monir Morshed, Ziyuan Li, Benjamin C. Olbricht, Lan Fu, Ahasanul Haque, Li Li, Ahmmed A. Rifat, Mohsen Rahmani, Andrey E. Miroshnichenko, Haroldo T. Hattori

**Affiliations:** 1 School of Engineering and Information Technology, University of New South Wales, Canberra, ACT 2610 Australia; 2 Department of Electronic Materials Engineering, Research School of Physics and Engineering, The Australian National University, Canberra, ACT 2601 Australia; 3 Coupled Optics LLC, 36 Wenark Drive, Delaware, Nework 19713 USA; 4 Australian National Fabrication Facility, The Australian National University, Canberra, ACT 2601 Australia; 5 Nonlinear Physics Centre, Research School of Physics and Engineering, The Australian National University, Acton, Canberra 2601 Australia

**Keywords:** Thermoelectric devices and materials, Nanoparticles

## Abstract

Nano-antennas are replicas of antennas that operate at radio-frequencies, but with considerably smaller dimensions when compared with their radio frequency counterparts. Noble metals based nano-antennas have the ability to enhance photoinduced phenomena such as localized electric fields, therefore-they have been used in various applications ranging from optical sensing and imaging to performance improvement of solar cells. However, such nano-structures can be damaged in high power applications such as heat resisted magnetic recording, solar thermo-photovoltaics and nano-scale heat transfer systems. Having a small footprint, nano-antennas cannot handle high fluences (energy density per unit area) and are subject to being damaged at adequately high power (some antennas can handle just a few milliwatts). In addition, given that nano-antennas are passive devices driven by external light sources, the potential damage of the antennas limits their use with high power lasers: this liability can be overcome by employing materials with high melting points such as chromium (Cr) and tungsten (W). In this article, we fabricate chromium and tungsten nano-antennas and demonstrate that they can handle 110 and 300 times higher fluence than that of gold (Au) counterpart, while the electric field enhancement is not significantly reduced.

## Introduction

The invention of radio antennas opened new opportunities to transmit information through free space. In recent years, very small replicas of radio antennas were invented at optical frequencies, so called nano-antennas. These antennas can boost the interaction of light with nano-scale matters by coupling and localizing the freely propagating visible and infrared optical radiation in a sub-wavelength region^1–3^. Key properties of the devices include localization and confinement of light below the diffraction limit^4^ and high electric field enhancement, which are generated by the excitation of localized surface plasmon resonances (LSPRs)^5,6^. These unique characteristics unlock a vast potential for different applications such as single molecule detection^7^, enhancement of the efficiency of photo-detection^8^, near-field optical trapping^9^, creation of nanoscale light sources^10^, solar energy harvesting^11^, heat transfer^12^, single algae cell detection^13^, surface-enhanced infrared absorption (SEIRA)^14^ and enhancement of surface enhanced Raman spectroscopy (SERS) signals^15^. The excitation of antennas is a non-resonant effect that allows the enhancement of the electric field at its extremities by exploiting the current appearing at the antenna surface and the lightning rod-effect. In general, electromagnetic waves in a large spectral range can be coupled to the antenna-however, it is a non-resonant coupling^1^.

However, the intraband transition of metals can lead to large photon absorption and, consequently, increase the local temperature of the antennas when they are irradiated by external laser sources^16,17^. Although the generation of localized heat regions in the antenna (hot-spots) may be useful for the treatment of cancer^18^, heat transfer^12^, and hot vapor generation^19^, the accumulation of heating and relaxation processes can produce unexpected effects on some applications such as imaging, sensing, and spectroscopy^20^. For example, the performance of the nano-antennas can be affected by the melting of the devices, resulting in a change of their morphology^21^. Therefore, the resonance may be either blue or red shifted, with significantly reduced electric field enhancement^22,23^. Given their small footprints, even small amounts of power can destroy nano-antennas since they have low thermal emittance in the mid to far infrared wavelength range^20^.

To overcome the above constraints, new materials are in demand for antennas to work under harder conditions. Guler *et al*.^24^ said that refractory plasmonic materials could be used to replace gold or silver in high power laser applications. Moreover, Mironov *et al*.^25^ stated that a proper selection of materials can help to create devices that can work with high fluences without melting. In recent years, distinctive typologies of nano-antennas are created such as dipole^26^, bowtie^4^, Yagi-Uda^27^, spiral^28^, and log-periodic antennas^28^ based on the gold material. However, the gold antennas can melt at a few microwatts of power or low fluence (e.g. 250 *μW* power or 0.059 *J*/*m*^2^ fluence)^25^. Therefore, new materials that can operate under higher fluences are needed to allow nano-antennas to work with high power lasers.

In this article, we demonstrate the performance of the bowtie nano-antennas made of different metals: gold (Au), chromium (Cr) and tungsten (W) under high fluences. Bowtie nano-antennas are chosen because these nano-antennas have high electric field confinement and enhancement due to the near-field coupling across the gap^5,29^. In addition, they are suitable for broadband operation^28^ and single molecule detection^7^. Our experiments show that chromium and tungsten antennas are capable of handling about 110 and 300 times higher fluence than that of the gold counterpart, respectively.

## Results

### Description of the structures

**Figure 1:**
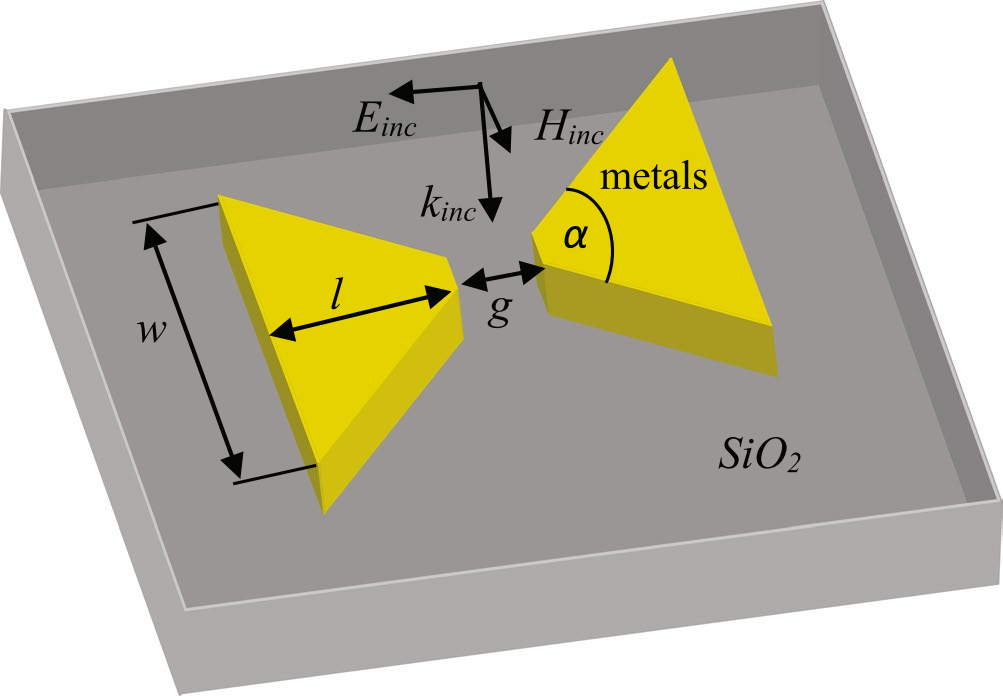
Schematic diagram of single bow-tie nano-antenna.

To compare how nano-antennas fabricated from different materials work at high fluences, a standard bow-tie shape is chosen for all materials. The antennas are fabricated on top of a quartz (*SiO*_2_) substrate, as shown in Fig. 1. The geometrical parameters of the antennas are as follows: the length of each trapezoid is *l*, width of the trapezoid is *w*, apex angle is *α*, and the gap width between two trapezoid is *g*. The yellow parts indicate metallic regions, which may be gold, chromium or tungsten, while the substrate material is silica in all cases. The electric field enhancement factor and absorption of the nano-antennas are calculated by optimizing the parameters at the wavelength of 1053 *nm*. The optimum length for gold, chromium, and tungsten nano-antennas are 190 *nm*, 145 *nm*, and 130 *nm*, respectively. The gap width, thickness and apex angle are always fixed to be 50 *nm*, 100 *nm* and 90° for antennas, respectively. It is noted that the electric field enhancement of the antenna can increase by reducing the gap size^25^.

### Electric field enhancement and absorption by nano-antennas

**Figure 2:**
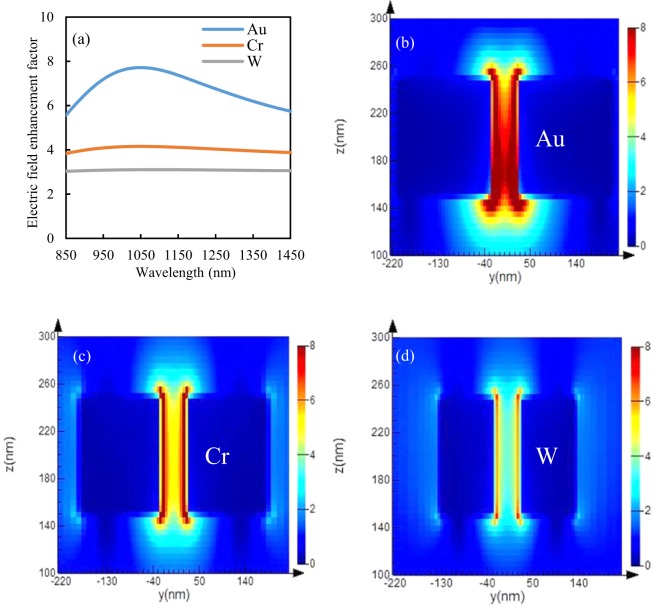
The electric field enhancement factor of (**a**) gold for the optimum parameters of *l* = 190 *nm*, and *w* = 380 *nm*, chromium for the optimum parameters of *l* = 145 *nm*, and *w* = 290 *nm*, and tungsten for the optimum parameters of *l* = 130 *nm*, and *w* = 260 *nm*, while the gap width *g*, thickness *H* and apex angle *α* are always kept constant to 50 *nm*, 100 *nm* and 90° for all nano-antennas and electric field intensity profile in the *y* − *z* (*E*_*inc*_ − *k*_*inc*_) plane of (**b**) gold, (**c**) chromium and (**d**) tungsten nano-antennas for the optimized parameters at the wavelength of 1053 *nm*.

Firstly, an finite-difference time-domain (FDTD) method is used to numerically calculate the electric field enhancement factor and absorption of the antennas with the optimized parameters (please see the Methods section). Figure 2(a) shows the electric field enhancement factor for all the three types of antennas at the optimum parameters and electric field intensity distribution along the vertical direction of nano-antennas are illustrated in Fig. 2(b–d) at the wavelength of 1053 *nm*. Figure 2(a) shows that the electric field enhancement at sub-wavelength gap of the gold bowtie antenna is relatively higher than the other two materials due to combined effects of this material-localized surface plasmon mode and gap plasmon mode. In addition, from Fig. 2(b), it is observed that the electric field is not uniform for gold antennas because much more light is concentrated at the interface between gold and silica due to larger refractive index compare to air. Whereas, in case of Cr and W, the two types of modes are also present-however, the surface plasmon resonance or resonance effect is reduced due to the high losses in both materials. This is confirmed by the electric field profiles of Fig. 2(c,d), where the electric field enhancement is weaker and quasi-constant across the gap between both nano-antennas. From the zoomed version of these figures we can see that the resonance exists but with a significant lower quality factor due to the metal losses. It is noted that, the electric field enhancement factor (calculated from Eq. 1) for gold, chromium, and tungsten are 7.5, 4.15, and 3.10, respectively. Since both tungsten and chromium absorb more power than reflective gold, the electric field is more uniform for chromium and significantly more uniform for tungsten when compared with gold, resulting in better uniformity of energy distribution in chromium and tungsten. The more uniform distribution of energy in the chromium and tungsten based nano-antennas can more uniformly distribute the temperature rise in the antennas.

**Figure 3:**
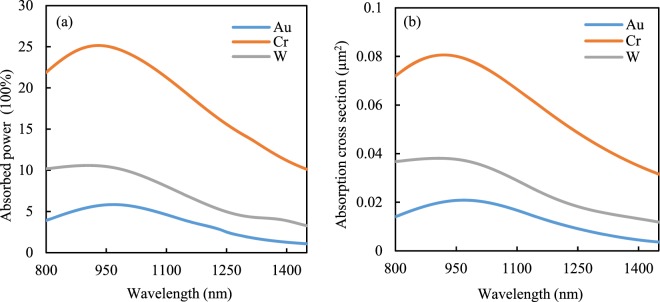
(**a**) Comparison of the absorbed power (heat genereation) and (**b**) numerically calculated absorption cross-section for three different nano-antennas.

One of the main problems in the field of thermo-plasmonics is that the continuous laser illumination induces temperature rise in the nano-antennas, which could eventually destroy the nano-antennas. The electromagnetic wave from the laser that strikes the metals in the nano-antennas can be converted into heat due to absorption of light or increasing of current density within the antenna^30^. The generation of heat in the nano-antennas depends on the physical properties of materials such as absorption that is related to the imaginary part of the refractive index^31^. To better understand the thermal behavior of these antennas, we have calculated the absorption of power and absorption cross section using Lumerical FDTD software which are shown in Fig. 3. Figure 3(a) shows the absorptance as a function of wavelength while Fig. 3(b) shows the absorption cross-section area as a function of wavelength. Although the figures show a peak at 950 *nm*, which differs from the peak of the electric field in the gap of nano-antenna because the energy distribution is not uniform along the plane of the antenna and while the absorptance is lower at 1053 nm, the electric field at the antenna edges is the highest which is clear from Fig. 2(b–d). We also observe from this figure that chromium has higher absorption than the other two materials at the wavelength of 1053 *nm* because chromium has comparatively higher imaginary dielectric constants at this wavelength^32–34^. In addition, chromium has lower thermal conductivity^35^ than gold and tungsten-therefore, it will generally produce more heat than gold and tungsten. It should be noted that the adhesive layer titanium can boost-up the temperature in the structures (even for a 2 nm thickness)^36^, i.e. it can add significant amount of heat to our antennas. However, in terms of damage, the antennas shape will not be changed until temperature rise in the metals exceeds their Tamman temperature. When the temperature in the surface of antennas higher than the Tamman temperature, the atoms diffusion and mobility increases significantly, resulting sintering or morphology changing of antennas^37,38^. The bulk melting points for gold, chromium, and tungsten is 1064.18 °*C*, 1907 °*C*, and 3422 °*C*, respectively. It is true that dielectric based antennas could reduce the power absorption by the antennas, but they generally have lower electric field enhancement^39^ and, in some applications, the nano-structures such as nano-antenna are used to convert optical power into heat^12^.

**Figure 4:**
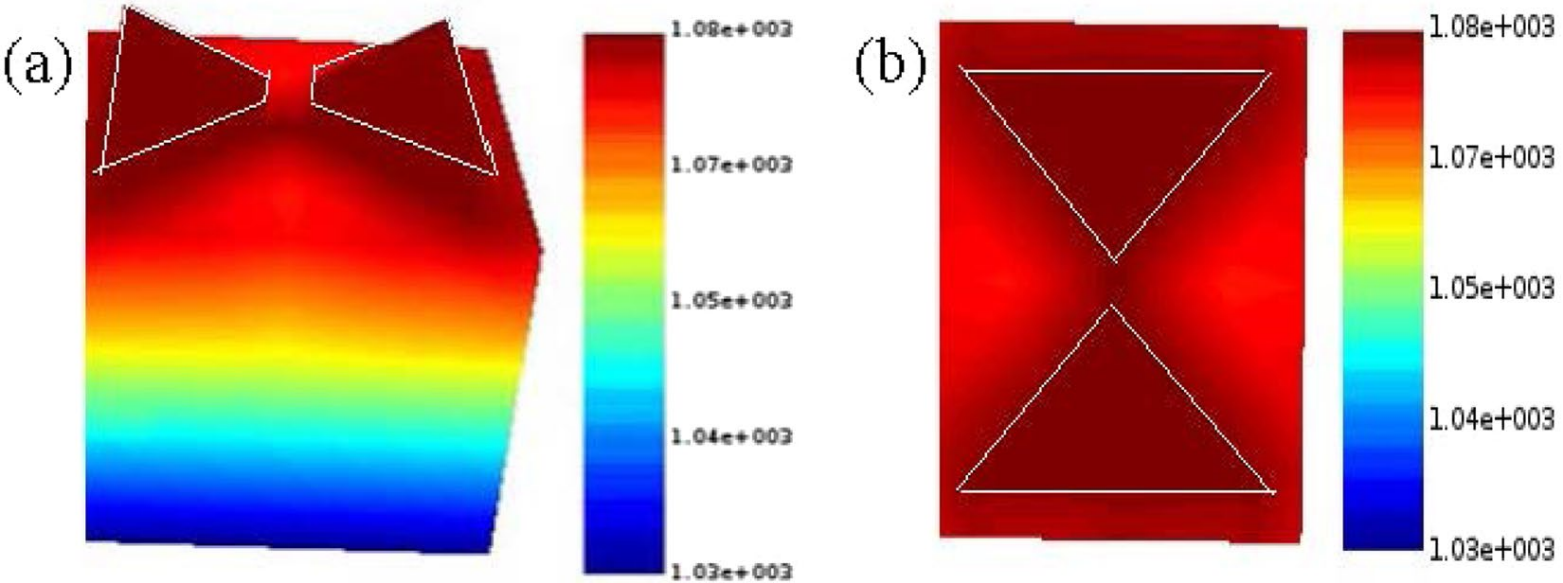
(**a**) 3D and (**b**) 2D view of the temperature distribution in gold nano-antennas at the power of 150 *μW*.

The main factor that leads to the melting of the metallic regions is the energy density or fluence^25,30,40^. When light reaches the antenna, heat is produced at the metal surfaces from the current density induced by light. In our experiments, we shall consider the fluence as the key parameter that can damage the nano-antennas. The laser fluence is calculated from the equations explained in the Methods section. Furthermore, we have analyzed the structures using the Lumerical device software package to see the thermal distribution in the nano-antennas, and as an example the distribution of temperature for gold antenna is presented in Fig. 4. Coppens *et al*.^30^ have also investigated the temperature distribution in nano-structures using the FDTD based software and claimed good approximation with the experiments.

For any fluence, the temperature is higher in the metallic and gap regions that than of the surrounding air and quartz substrate as can be observed in Fig. 4. It is because the thermal conductivity of air (0.0257 *W*.*m*^−1^.*K*^−1^) and Quartz (1.3 *W*.*m*^−1^.*K*^−1^)) is comparatively low-so that, the heat generated by nano-antennas cannot totally dissipate, therefore, they concentrates in the antennas region.

### Fabrication and characterization of nano-antennas

**Figure 5:**
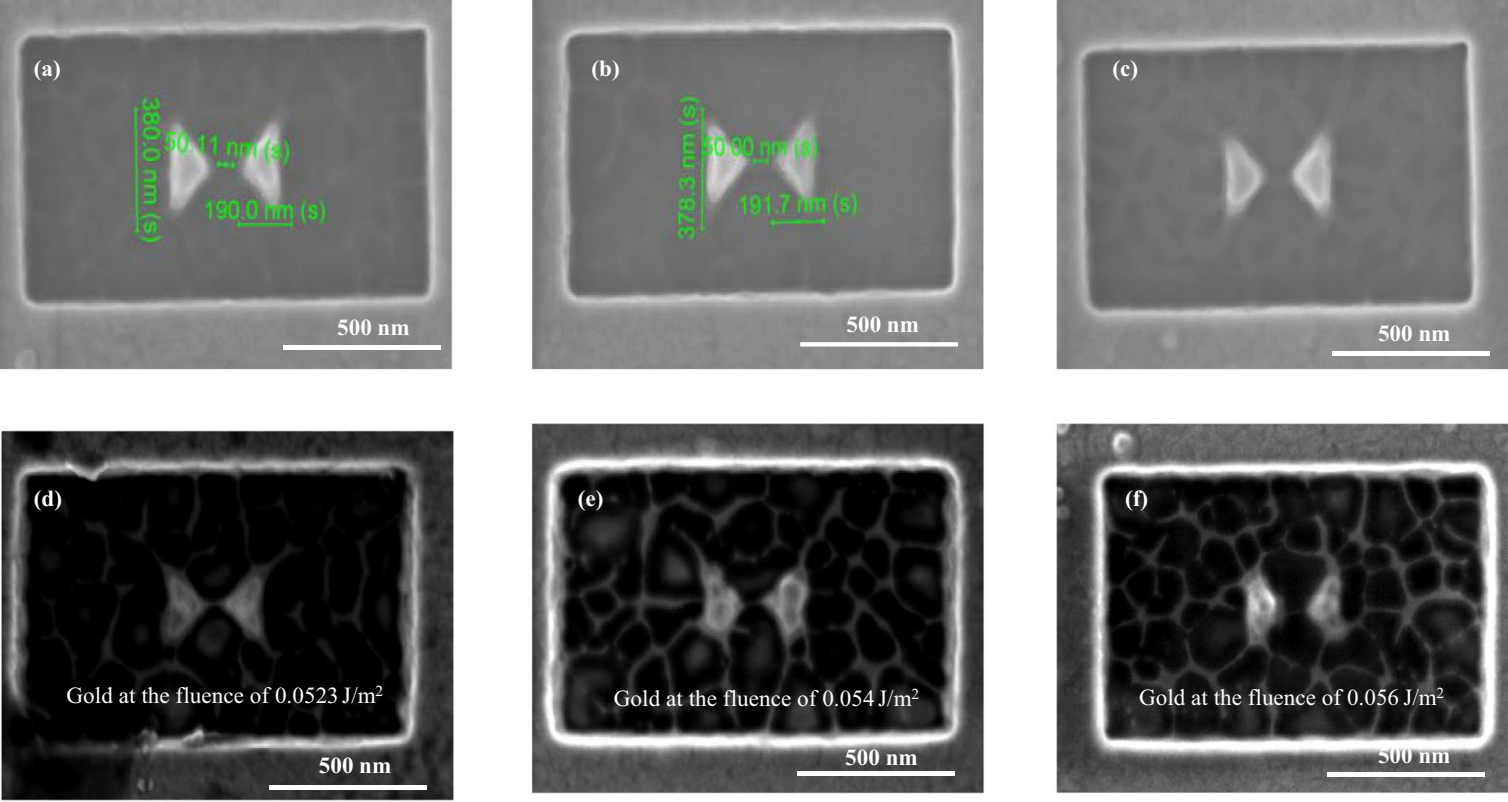
SEM images of the studied gold nano-antennas (**a**–**c**) without laser exposure and (**d**–**f**) under the fluence of 0.0523 *J*/*m*^2^, 0.054 *J*/*m*^2^, and 0.056 *J*/*m*^2^, respectively which clearly indicates the melting of nano-antennas.

The antennas are fabricated by firstly depositing different metals using electron-beam evaporator or sputter system and the patterns are made by focused ion beam(FIB) as described in the Methods section. Firstly, the gold bow-tie antenna is fabricated and characterized, which is shown in Fig. 5. Figure 5(a–c) shows SEM images of a gold bowtie nano-antennas with no laser exposure. In order to examine the damage threshold, the antennas are exposed to different fluences: we start from a low fluence of 0.01 *J*/*m*^2^, then we increase the fluence by 0.012 *J*/*m*^2^ (step size) until the gold antenna is damaged. From Fig. 5(d) it shows that the antenna starts to damage at the fluence of 0.0523 *J*/*m*^2^, However- they are completely damaged for the fluence of 0.054 *J*/*m*^2^ and 0.056 *J*/*m*^2^, as shown in Fig. 5(e,f). Besides that, the cumulative heating which is raised from the absorption of power may lead to the shape deformation when the melting temperature is exceeded. We also observe some surface damaging because we used 4 nm Ti layer as a adhesive layer that may also be damaged at threshold fluence of gold.

**Figure 6:**
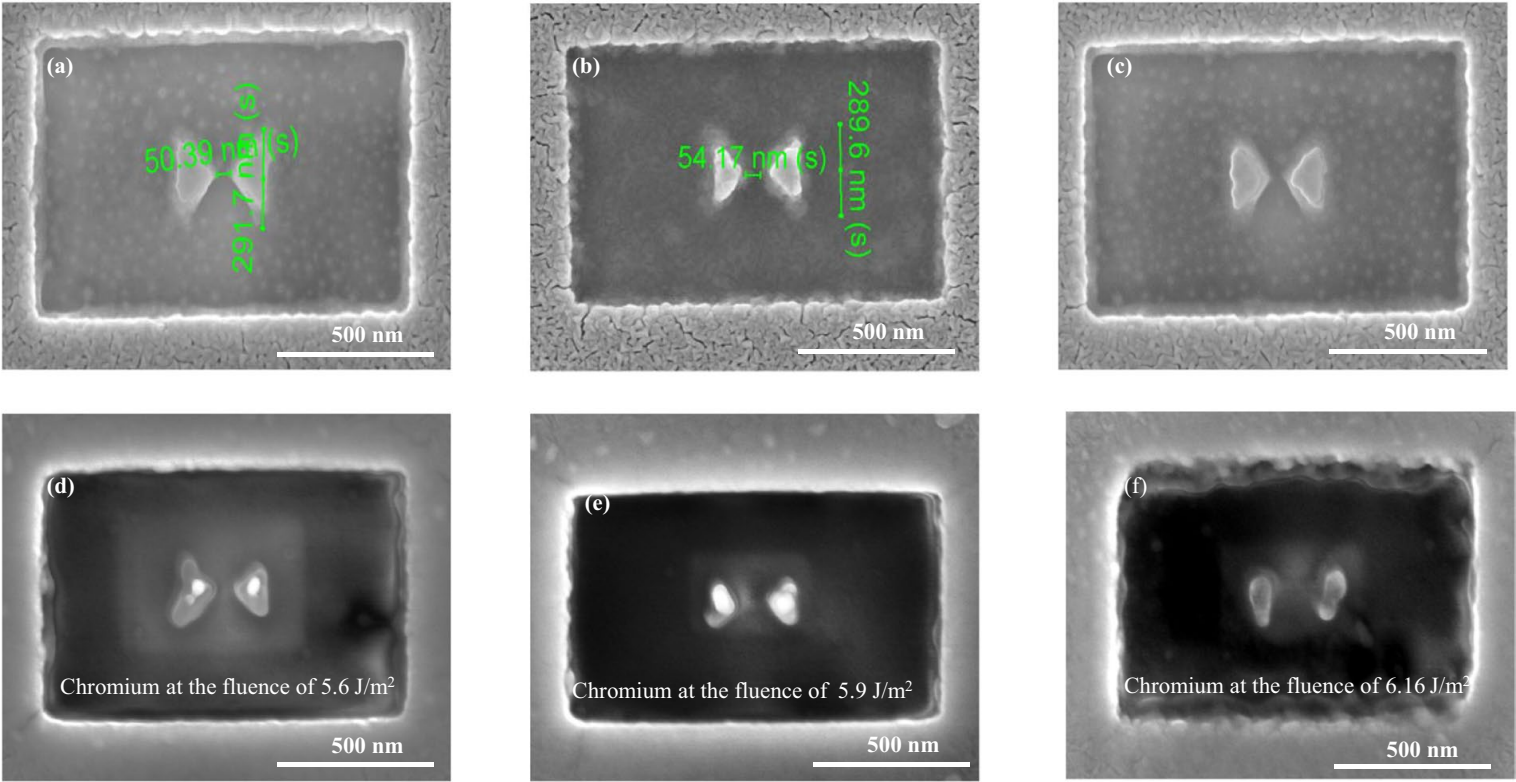
SEM images of the studied chromium nano-antennas (**a**–**c**) without laser exposure and (**d**–**f**) under the fluence of 5.6 *J*/*m*^2^, 5.9 *J*/*m*^2^, and 6.16 *J*/*m*^2^, respectively, which clearly indicates the melting of nano-antennas.

**Figure 7:**
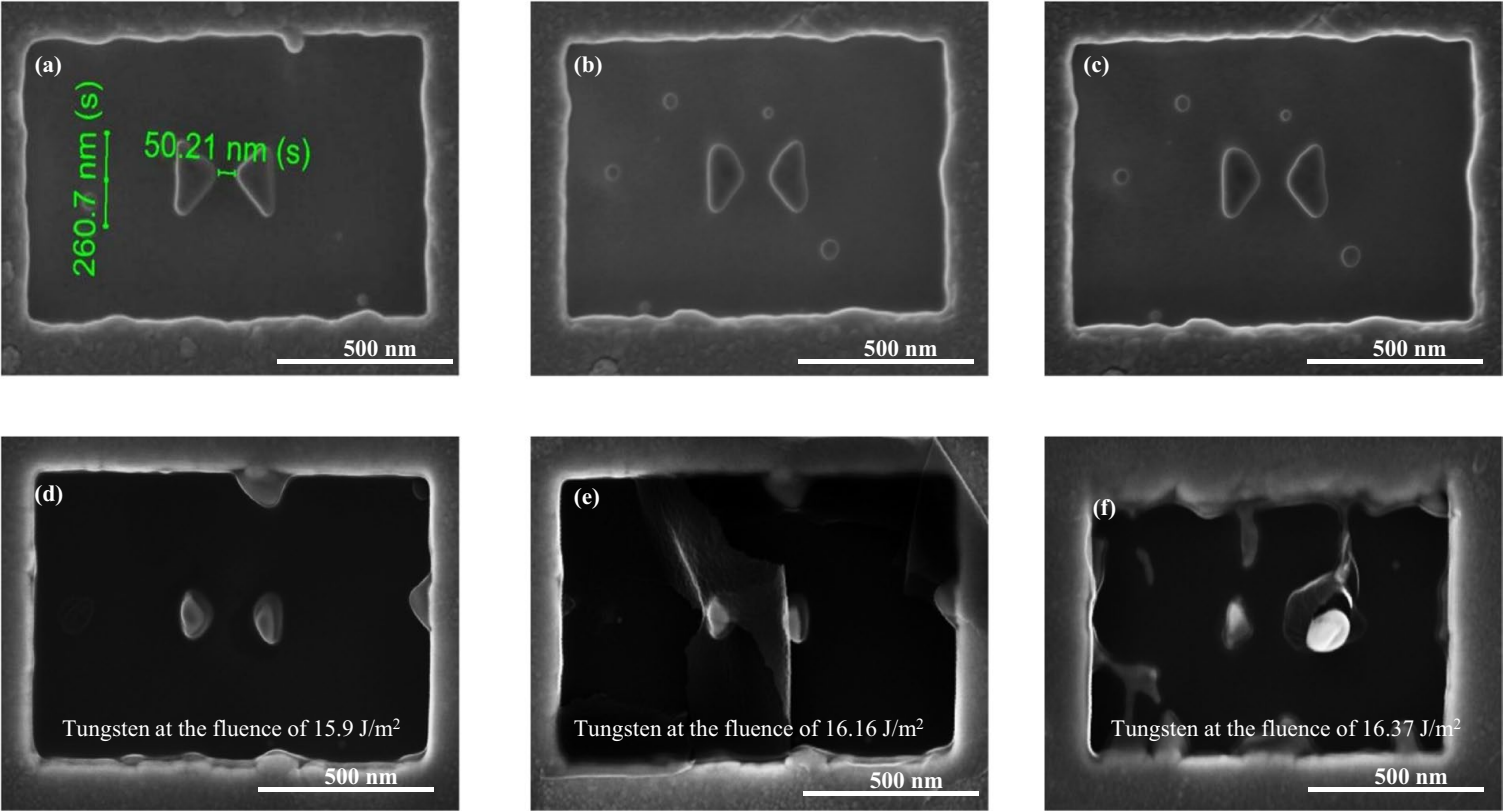
SEM images of the studied tungsten nano-antennas (**a**–**c**) without laser exposure and (**d**–**f**) under the fluence of 15.9 *J*/*m*^2^, 16.16 *J*/*m*^2^, and 16.37 *J*/*m*^2^, respectively which clearly indicating melting of nano-antennas.

We have also fabricated chromium and tungsten nano-antennas, which are shown in Figs 6(a–c) and 7(a–c). The SEM images show that the shapes of the fabricated antennas are not as well-defined as the gold counterparts. The main reason is that chromium and tungsten are both hard materials which are harder to remove by the Gallium ions. The contamination of Ga ions may increase some losses and thus lead to a broadening of the resonance. However, the effect is minor and will not affect much the near field behaviour of the antennas^41^. The antennas geometry can be optimized by using the electron beam lithography (EBL) technique and related lift-off techniques. Nevertheless, the antennas presented in this work are sufficient to be used for the laser fluence test.

We start the exposure of chromium from the gold threshold fluence of 0.054 *J*/*m*^2^, and increase the fluence until the antenna is damaged. The devastated chromium nano-antennas are appeared in Fig. 6(d–f) and the fluence of 6.16 *J*/*m*^2^ is considered as the threshold damage fleunce for chromium. we can also see from this figure that the metallic regions are mostly damaged due to the higher absorptance of chromium.

Lastly, we apply different fluences for tungsten nano-antennas starting from the chromium threshold damage fluence as a reference. The fluence that damages the antennas is about 16.16 *J*/*m*^2^. Figure 7(d–f) shows the SEM image for the damaged antenna. This figure shows that the antenna starts to melt at a fluence of 15.9 *J*/*m*^2^, and is completely damaged at the fluence of 16.16 *J*/*m*^2^ and 16.37 *J*/*m*^2^, respectively. Now, the threshold fluence of tungsten antenna is considered as 16.16 *J*/*m*^2^.

## Discussion

From the above results, we can see that the fluences for gold, chromuim, and tungsten are 0.054 *J*/*m*^2^, 6.16 *J*/*m*^2^, and 16.16 *J*/*m*^2^, respectively. Therefore, in comparison with gold and chromium counterparts, tungstens threshold damage fluence is 2.73 times and 300 times higher than that of chromium and gold antennas, respectively. In addition, chromium and tungsten has 6 and 7.22 times higher magnitude of electric field intensity in the gap than that of gold counterpart when they operate at their threshold fluence. Since, the incident electric field *E*_*inc*_ is proportional to the square root of the fluence of a laser where, spot size and repetition rate are constant, so, we can easily calculate the magnitude of electric field intensity in the gap for different antennas from Eq. 1 (see Methods) for given electric field enhancement factors. The possible reason behind the improved performance of tungsten antenna could be its lower absorbance (see Fig. 3), higher thermal conductivity than chromium and significantly higher melting point. Moreover, since tungsten has higher dissipative loss than gold, more light penetrates into the metal, therefore less power is localized on the surface of tungsten nano-antennas. Also, we can clearly see from Fig. 2(c) that the electric field is uniformly distributed in the gap. Although gold is more reflective and less absorbing than tungsten, tungsten has almost 3.2 times than gold’s melting point.

**Figure 8:**
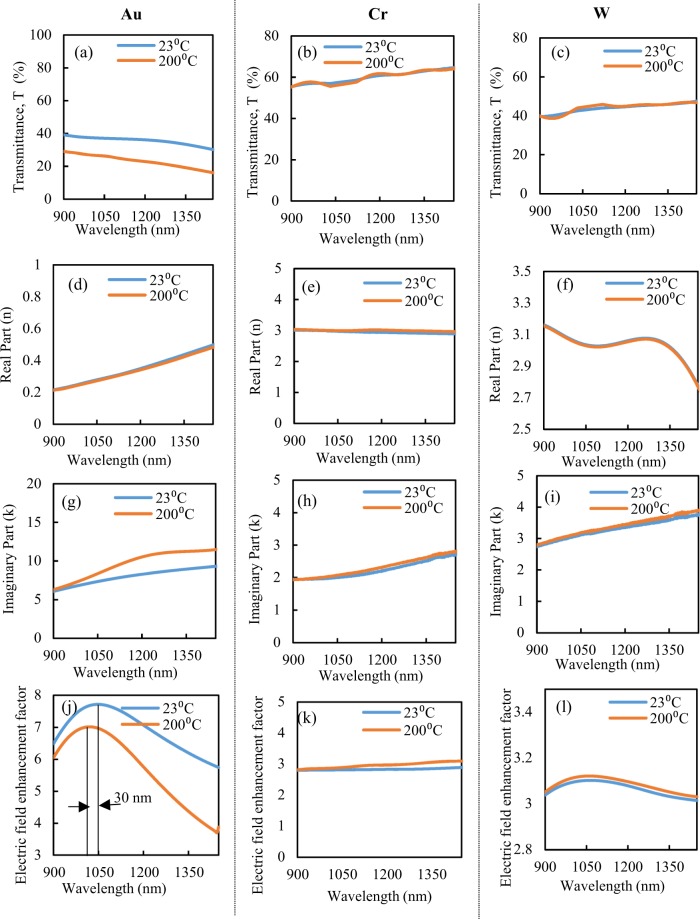
Experimentally optical properties measurement of gold, chromium and tungsten, respectively for different temperatures: (**a**–**c**) transmission, (**d**–**f**) real part of index, (**g**–**i**) imaginary part of index, (**j**–**l**) electric field enhancement factor calculated from experimental data.

The temperature on the antenna surface is one of the most important parameters that eventually lead to the destruction of the devices. To better understand the temperature dependence of the optical properties of different materials and how they affect the electric field enhancement factors, we have used another setup (see Methods section). The transmission and refractive indices of gold, chromium and tungsten thin films at different temperatures are shown in Fig. 8(a–c), and (d–i) respectively. From Fig. 8(a), it is observed that the transmission of the gold film decreases with increased temperature and, from Fig. 8(d,g), it can be inferred that, although the real part of the refractive index does not change much, the imaginary part increases significantly with increased temperature. Therefore, the absorption of films can increase at high temperatures. We have also calculated the electric field enhancement factors based on the measured temperature dependent refractive indices as shown in Fig. 8(j), which match the theoretically simulated results, confirming the accuracy of our calculations. In Fig. 8(j), it is clear that the electric field enhancement decreases, and the resonance blue-shifts with increased temperature. For chromium, it can be observed, from Fig. 8(b,e,h) that the transmission and refractive indices change slightly with temperature. Similarly, the properties of tungsten also change slightly with temperature. Moreover, the electric field spectra have no resonance shift (Fig. 8(l)). Therefore, we may conclude that tungsten is more stable than gold and chromium at high temperatures, and can operate at high powers when compared with the other materials. It is noted that the nano-structures continuously heat up when irradiated by external laser sources: the electric field intensity and dissipated power increase the temperature of nano-antennas^42,43^.

In the above paragraph, we have just explained indirect way to measure the electric field enhancement factor of different nano-antennas and how they may be affected by the generation of heat from high power. Although some research groups have measured the electric field profile of nano-antennas^4^, they have stated that it was very difficult to accurately measure the electric field intensity in the antennas since the probe (e.g. near-field scanning optical microscopy (NSOM) probe) would strongly interfere with the measurement at the nanometer scale, making their measurements rather qualitative.

Some groups have simulated the rise of temperature in nano-antennas^43^ and they have shown that the temperature really modifies the electric field enhancement and absorption of plasmonic devices. In another theoretical paper by Downes *et al*.^42^, they stated that NSOM could detect scattered power with atomic resolution, but very thin tips (less than 30 nm diameter) would be needed. The measurement of local temperature at nanoscale remains difficult because the tips are generally much larger than the nano-device, meaning that the measured temperature is the average temperature of a much larger areas than the device. Based on the reports, the local temperature in nano-antennas and the local electric field enhancement remain hard to be directly measured.

We have indirectly measured the electric field enhancement of nano-antennas by using Raman spectroscopy (the Raman intensity is proportional to the fourth power of the electric field), but only at room temperature^44^. The Raman equipment used has an internal laser and optical parts that could be damaged at high temperatures and, moreover, the equipment does not have enough room to accommodate heaters to control the temperature of the antennas.

## Conclusions

In outline, for high power laser operation, we have proposed a new methodology to design high fluence bowtie nano-antennas based on diverse materials such as gold, chromium and tungsten. The results show that in spite of the fact that tungsten have 58.7% lower electric field enhancement than gold under the same laser excitation, it can work at 300 times higher fluence as well as tungsten can accomplish 7.22 times higher magnitude of electric field than that of gold counterpart when they work at their threshold fluences-therefore, they may be valuable to work with high power lasers. Moreover, we measured the optical properties for gold, chromium and tungsten at 200 °*C* temperature and our result shows that the real part of index for all materials remain almost intact, however, the imaginary part for gold changes dramatically, and for tungsten still shows almost similar value to that at room temperature. Therefore, tungsten may perform more stably under high power laser application compared with gold.

## Methods

The numerical analyses of the optical properties of our nano-antennas are conducted by using commercial three-dimensional FDTD software^45^ with perfectly matched layer boundary conditions. We have used three different materials such as gold, chromium, and tungsten in our nano-antennas. In the simulations for Figs 2 and 3, the refractive indices of different materials were set by using the Lumerical software’s default data, while for Fig. 8 the indices were measured by using an ellipsometer at different temperatures and used as an input parameter to the Lumerical FDTD software. The whole structure is surrounded by air and the incident wave is assumed to be a quasi-plane wave. The spot size diameter of the source is much larger than the computational area of the structure. The mesh size is chosen as Δ*x* = Δ*y* = Δ*z* = 5 *nm*. Since electric field in the gap of nano-antenna is enhanced, the relative electric field enhancement factor can be calculated as,

1 $${F}_{rel}=\frac{|{E}_{gap,peak}|}{|{E}_{inc,peak}|}$$

where, |*E*_*gap*,*peak*_| is the magnitude of the electric field calculated in subwavelength gap of the nano-antenna, and |*E*_*inc*,*peak*_| is the magnitude of electric field of the incoming light.

The laser fluence is calculated from the following equations.The energy and fluence for a single pulse of a Q-switched laser are given by^25^,

2 $${W}_{single-pulse}={\int }_{{t}_{1}}^{{t}_{2}}\,P(t)dt={P}_{peak}{\tau }_{eff}$$

3 $${F}_{single-pulse}={W}_{single-pulse}\frac{4}{\pi {r}_{spot}^{2}}$$

where, *P*(*t*) is the instantaneous value of the power, *t*_1_ and *t*_2_ are the arbitrary instants when the pulse is not negligible, *τ*_*eff*_ is the effective duration of the pulse, and *r*_*spot*_ represent the spot size of the laser beam.

The antennas were fabricated by firstly depositing different metals using electron-beam evaporator or sputter system. The thickness of the metals in each of the three samples was taken as 100 nm and for the gold sample, the metal was deposited by Temescal BJD-2000 E-beam/Thermal Evaporator system at the rate of 5 *nm*/*s*. A 2 *nm* titanium layer was used to provide good adhesion between gold and the substrate. On the other hand, the chromium and tungsten were deposited by Sputter Coater system-*AJA*. However, the deposition rate of the metals was different: 2.58 *nm*/*min* and 6 *nm*/*min* for Chromium and Tungsten, respectively. For tungsten Ti was used as adhesion layer to the substrate, whereas for chromium due to its good adhesive property, no titanium adhesion layer was used. The nano-antenna patterns were milled by FEI Helios NanoLab 600 dual beam focused ion beam (FIB) system. All the scanning electron microscope (SEM) images were taken using either FIB or Electron Beam Lithography (EBL-Raith 150) system.

**Figure 9:**
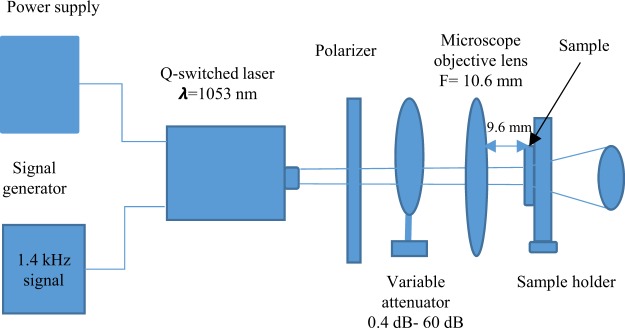
The schematic diagram of the laser setup for the characterization process of nano-antennas.

For the characterization of our devices, a Lastek Q-switched commercial laser with pulse duration of 10 *ns* was used, which operates at the wavelength of 1053 nm. Generally, the spot size of the laser is 2 mm with a maximum energy of 50 *μJ* and the calculated maximum fluence of this laser is 0.785 *J*/*m*^2^. However, the spot diameter of laser can be changed using Olympus 10x infinity plan microscope objective lens that helps to increase the fluence (energy density). The estimated maximum fluence of this laser using the microscopic lens is 83.055 *J*/*m*^2^. The normal working distance of that lens is 10.6 mm, however-the light start ti diverge beyond this distance. In our experiment, we fixed the sample 9.6 mm away from lens. The whole characterization process is shown in Fig. 9, where a 1.4 *kHz* repetition rate signal is used.

**Figure 10:**
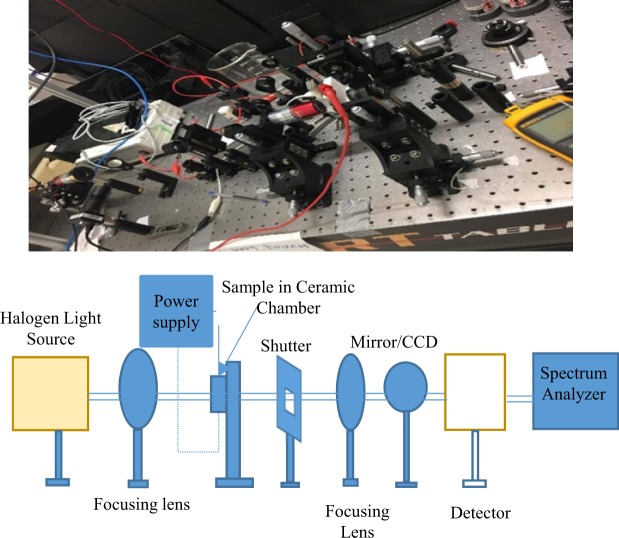
Schematic diagram of experimental setup to measure the transmission of thin film.

Finally, to assess how heat generation in nano-antennas can affect the electric field enhancement factor, we apply different temperatures with our home-made setup and measure the temperature dependent optical properties of the metals. We have used two different setups. To measure the transmission, a home-built white-light spectroscopy setup in confocal configuration is used which is shown in Fig. 10. The sample is illuminated by a white-light source (fiber coupled tungsten halogen light bulb) and an infrared (IR) spectrometer is used. For transmission spectra measurement at different temperatures, a Mitutoyo M Plan NIR 10x NA 0.26 objective is used, with the light transmitted through the sample being measured at different temperatures, then collected and directed to the spectrometer. A modified home-made heater with two electrodes is used control the sample temperatures. To perform this experiment 15 nm film thickness is used for all metals, which are deposited by a sputter system. The temperature in the surface of thin films is measured by an IR thermo-meter. On the other hand the refractive indices at different temperatures are measured using a Variable Angle Spectroscopic Ellipsometer (VASE). It is noted that in order to apply various temperatures in the sample under the same condition while measuring the optical properties, a same voltage dependent heater is mounted onto the VASE. Then, these experimental data (refractive indices) for three materials are used as input parameters to Lumerical FDTD software to calculate the electric field enhancement factor.
